# Changes in Physical Fitness, Muscle Damage and Cognitive Function in Elite Rugby Players over a Season

**DOI:** 10.3390/sports12080223

**Published:** 2024-08-16

**Authors:** Mohamed Houssem Karamti, Hassane Zouhal, Mariem Bousselmi, Manel Darragi, Hamdi Khannous, Ahlem Ben Hmid, Imen Zamali, Mélika Ben Ahmed, Ismail Laher, Urs Granacher, Amira Zouita Ben Moussa

**Affiliations:** 1 Higher Institute of Sport and Physical Education of SFAX, University of Sfax, Sfax 3027, Tunisia; houssemkaramti@hotmail.com (M.H.K.); b.mariem@yahoo.fr (M.B.);; 2 Research Laboratory (LR23JS01) “Sport Performance, Health & Society”, Higher Institute of Sport and Physical Education of Ksar Said, Tunis 1000, Tunisia; 3 Movement, Sport, Health and Sciences Laboratory (M2S), UFR APS, University of Rennes 2-ENS Cachan, Av. Charles Tillon, CEDEX, 35044 Rennes, France; 4 Institut International des Sciences du Sport (2I2S), 35850 Irodouer, France; 5 Tunisian Rugby Federation, Tunis 1000, Tunisia; 6 Clinical Immunology Department, Pasteur Institute of Tunis, Tunis 1000, Tunisia; ahlem.benhmid@fmt.utm.tn (A.B.H.);; 7 Laboratory of Transmission, Control and Immunobiology of Infections (LR11IPT02), Pasteur Institute of Tunis, Tunis 1000, Tunisia; 8 Faculty of Medicine of Tunis, Tunis El Manar University, Tunis 1000, Tunisia; 9 Department of Anesthesiology, Pharmacology and Therapeutics, The University of British Columbia, Vancouver, BC V6T1Z4, Canada; 10 Department of Sport and Sport Science, Exercise and Human Movement Science, University of Freiburg, 79102 Freiburg, Germany; 11 Higher Institute of Sport and Physical Education of Ksar Said, University of Manouba, Tunis 1000, Tunisia

**Keywords:** BDNF, elite rugby players, longitudinal monitoring, muscle damage, physical performance

## Abstract

This study proposes to monitor the physical, immune and cognitive responses and adaptations of elite rugby players throughout the season based on the loads performed. Anthropometric measurements, physical fitness tests (e.g., muscle strength and power, linear and change-of-direction speed, cardiorespiratory fitness) and analyses of serum concentrations of markers of muscle damage (creatine kinase [CK] and lactate dehydrogenase [LDH]) and brain-derived neurotrophic factor (BDNF) were carried out over a sporting season (24 weeks) for 17 elite rugby players (10 forwards and 7 backs) aged 18.91 ± 0.76 years. The physical fitness test results show improvements in the performance of both forwards and backs over the season (*p* < 0.05), with an advantage for backs compared with forwards in most tests (*p* < 0.05). Muscle damage markers decreased at the end of the season compared with the baseline levels for forwards (*p* < 0.05). CK levels were unchanged for the backs, but there were increased LDH concentrations at the end of the season compared with baseline (*p* < 0.05). Serum BDNF levels decreased for the total group between the second and third sampling (*p* < 0.05). The muscular and physical capacities of rugby players differ according to their playing position. Immune responses and adaptations, as well as BDNF levels, vary throughout the season and depend on the physical load performed.

## 1. Introduction

The game of rugby has undergone a significant transformation, evolving into a fast-paced, dynamic sport with linked actions and more continuous play, particularly in elite rugby [1]. Rugby is an intermittent sport with periods of high intensity and intervals of low intensity. The evolution of players’ physical skills, which has resulted in athletes becoming bigger, stronger and faster, is linked to the game’s transformation [1].

Athlete monitoring has become a standard procedure in professional sports [2,3]. Measuring the physical, physiological and psychological demands during training and competition and the related long-term adaptations have become a priority in professional team sports (e.g., rugby) in recent years [4,5]. The most commonly assessed monitoring parameters in rugby involve measures of physical fitness, such as body composition and cardiorespiratory fitness, as well as linear and change-of-direction speed [6,7,8]. Previously, findings from cross-sectional studies indicate significant correlations between physical and physiological characteristics (e.g., body mass, muscular power and muscular strength) and match-play success and on-field rugby performance y [9,10,11]. The data obtained can help staff and performance managers optimize the training process throughout the season, enabling players to achieve high-performance levels [12,13].

The physical exertion inherent to the sport of rugby can result in fatigue and muscle damage, which can subsequently impact neuromuscular function and player performance [14,15]. In this context, the parameters creatine kinase (CK) and lactate dehydrogenase (LDH) can be employed to monitor muscle fatigue and damage in rugby players, thereby assisting coaches and technical staff to better program training with the goal of avoiding injuries and optimizing players’ performance [16].

The fluctuations in muscle damage markers observed throughout a sporting season are influenced by a multitude of factors, including the physical load imposed, the recovery protocols employed and the players’ capacity to adapt to the physical demands of the game [17].

Well-developed cognitive skills are important for success in rugby, as they can influence player performance and distinguish between different levels of play [18]. It is, therefore, important to understand the underlying mechanisms of cognitive function in response to exercise. Physical activity stimulates the release of neurotransmitters and neurotrophins, which clearly potentiate neuronal function and brain plasticity. Although a range of mechanisms contribute to brain plasticity, the intervention of brain-derived neurotrophic factor (BDNF) is crucial to exercise-induced brain plasticity and improved cognitive function [19]. BDNF is a protein of the neurotrophin family, synthesized mainly by the brain and peripheral tissues, such as adipose tissue, skeletal muscle and immune cells. This protein is closely linked to brain plasticity, neuronal survival and synapse formation and plays a potential role in enhancing cognitive functions, such as learning and memory [20]. Progress has been made in understanding the effect of exercise on BDNF and cognitive function. Changes in BDNF levels depend on the type, intensity and duration of physical effort [21]. Further studies are needed to shed more light on the dose–response relationship between physical activity and training with BDNF, particularly in professional athletes who require sophisticated cognitive skills for their success.

Rugby differs from other sports in the heterogeneity of its players’ anthropometric and physical characteristics depending on their playing position. These can be generalized into forwards and backs, comprising eight and seven players, respectively [22]. Forwards are involved in static phases, such as scrums and line-outs, and phases of conquest and ball conservation, such as rucks and mauls. In contrast, backs are open-field and evasive players, covering greater distances and performing higher frequencies of speed and change of direction than forwards [23,24,25]. 

The U20 international game has received much attention in recent years. Research on top-level teams participating in major international events focused on the physical development of U20 internationals. Players must possess a wide range of muscular and physical abilities and reach sophisticated levels to meet the demands of the game [8,26,27], which vary greatly depending on the position they occupy [25]. This underscores the importance of monitoring the evolution of physical and physiological skills in this age group, which could improve control and the orientation of training, the establishment of short- and long-term objectives and the preparation of these young players for the senior game [6]. However, there is a lack of knowledge regarding players’ physical profiles and levels of play in North African teams.

To develop the field of performance analysis in rugby, collaboration between scientists and practitioners is necessary to improve the ability of science to influence practice. The theory-to-practice gap may be bridged by the development of an applied research model that describes rugby performance in an integrated manner. Based on the above reasoning, the current study’s primary aim was to conduct an extensive longitudinal analysis (24 weeks) of physical fitness, markers of muscle damage and cognitive function among elite U20 rugby players. Monitoring was conducted (i) according to playing position and (ii) over three assessment sessions during a training period leading up to an international event. We hypothesized that physical performance, muscular damage markers and BDNF levels may be altered after long-term rugby training.

## 2. Materials and Methods

### 2.1. Participants

The minimum sample size was determined by calculating an a priori power analysis using G*Power (version 3.1, University of Düsseldorf, Germany). The power analysis was calculated with an assumed power of 0.90 and an alpha level of 0.01, a non-sphericity correction of 1 and a high effect size (Cohen’s f) of 0.97 taken from a related study for 10 m linear sprint speed (Freitas et al., 2018) [28]. The analysis revealed a total sample size of 8 participants. Therefore, we recruited additional players (*n* = 26) to allow for potential drop-outs. 

At the start of the study, we recruited 26 elite participants from the Tunisian national U20 15-a-side rugby team. During the season, a number of players suffered injuries and were unable to attend all scheduled training sessions, so they were excluded from the study. A total of 17 players were eligible for the study. This group of voluntary participants included ten forwards and seven backs. The average age of the players was 18.91 ± 0.76 years, and the number of years of practice was 7.41 ± 1.12 years. All participants were informed of the purpose of the research and gave their consent to participate in the entire study. The study was approved by the Ethics Committee of the University of Sfax, Tunisia, (CPP SUD No. 0497/2023), and the study procedures followed the latest version of the Declaration of Helsinki.

### 2.2. Procedures

**Figure 1:**
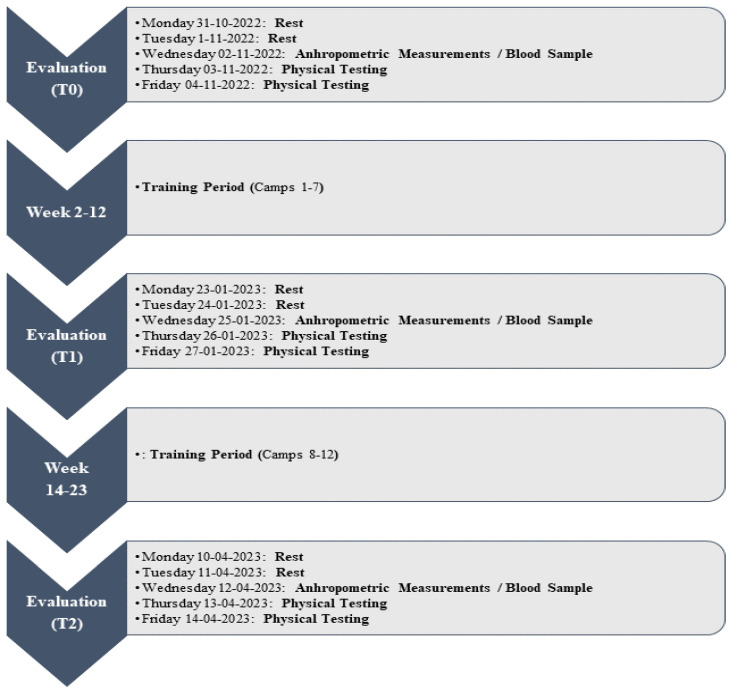
Experimental procedures: (T0): at the beginning, (T1): in the middle, and (T2): at the end of the season.

Longitudinal monitoring of the Tunisian U20 men’s 15-a-side rugby national team was carried out during the preparatory period for the U20 African Cup Barthés Trophy (Kenya, 22–30 April 2023). Preparation for the tournament lasted for 24 weeks (early November to mid-April), during which 12 training camps were held in Tunisia at the same venue that was affiliated with the Rugby Federation. Three evaluation sessions were carried out during this period: at the beginning (T0), in the middle (T1) and at the end of the season (T2). Assessments included anthropometric measurements, physical fitness tests and monitoring of muscle damage markers and changes in serum BDNF levels (Figure 1).

### 2.3. Anthropometric Measurements

Body mass and height were measured to the nearest 0.1 kg and 0.1 cm, respectively, and the body mass index (BMI) was calculated. After measuring the thickness of four skin folds (bicipital, tricipital, sub-scapular and supra-iliac) with a Harpenden caliper, the percentage of body fat mass (% BFM) was estimated according to the formula of Durnin and Womersley [29]. All measurements were taken in the morning by the same investigator, and players were wearing short trousers.

### 2.4. Physical Fitness Testing

The tests consisted of assessing maximum aerobic speed (the Yo-Yo Intermittent Recovery Test), linear speed (10 m, 20 m and 30 m), change of direction speed (*t*-test), lower limb power (countermovement jump and standing long jump) and maximum strength (squat, bench press and prone row).

#### 2.4.1. Cardiorespiratory Fitness

Cardiorespiratory fitness was evaluated with level 1 of the Yo-Yo Intermittent Recovery Test (YYIRT). Athletes completed two 20 m shuttles at progressively increasing speeds to the rhythm of a beeper. The round trips were interspersed with 10 s of jogging 5 m behind the finish line after each 40 m. The test ended when players could no longer maintain the set pace, and the last level achieved was recorded [7]. The intraclass correlation coefficient (ICC) for test–retest reliability was 0.948 (0.884–0.980). 

#### 2.4.2. Linear Sprint Speed

The evaluation of linear speed over 30 m and times were measured with photocells (Witty Timing System, Microgate, Balzano, Italy) positioned on the starting line and 10 m, 20 m and 30 m finishing lines. Players stood ~0.5 m behind the start line before commencing the speed test. Each athlete performed two maximal sprints separated by three minutes of passive recovery, and the best time was recorded [30]. The ICC for 10 m, 20 m and 30 m was 0.894, 0.924 and 0.915, respectively.

#### 2.4.3. Change-of Direction Speed

The assessment of change-of-direction speed was realized using a standardized *t*-test protocol. Each participant was required to sprint 9 m forward, perform a lateral displacement of 4.5 m to the left, change direction towards the right extremity and finish by returning to the middle and retreating to the starting point. Two trials separated by three minutes of passive recovery were performed by each participant, and the best time was taken as the *t*-test score [31]. The ICC of the test was 0.954. 

#### 2.4.4. Proxies of Lower Limb Muscle Power

Lower limb power was assessed using horizontal and vertical jumps. Each player was asked to perform two trials of the standing long jump (SLJ) and the countermovement jump (CMJ), separated by one minute of passive recovery after each jump.

##### SLJ

Each player was asked to perform long jumps from a standing position. Players were allowed to start the jump with bent knees and swing their arms to facilitate movement. A line drawn on the ground was used as the starting line. The distance covered was measured by a tape measure attached to the floor, and performances were measured 1 cm from the heel closest to the starting line [28]. The ICC of the test was 0.980.

##### CMJ

Each player assumed a standing position with a knee angle of 180°, performed downward countermovements until their knee angle was near 90° and then immediately jumped as high as possible [30]. Their maximal vertical jump performance was measured using an Optojump (Optojump, Microgate, Bolzano, Italy). The ICC of the test was r = 0.985.

#### 2.4.5. Muscle Strength

All players were experienced in these exercises and performed a warm-up with self-selected loads before the 1RM performances expressed in kg. The players performed three trials interspersed with 3 min of recovery for each test.

##### Back Squat

While maintaining a neutral back position and heels on the ground, participants were required to squat until their upper thigh was parallel to the ground and then return to the starting standing position [9]. The ICC of the test was 0.903.

##### Bench Press

The test consisted of lowering the bar to touch the chest, then lifting it until the elbows locked, keeping the shoulders and hips in contact with the bench and feet flat on the floor [9]. The ICC of the test was r = 0.760.

##### Prone Row

Each player lay on a bench facing the floor. The participant had to pull the bar until it touched the bench on both sides. The height of the bench was adjusted by the participant so that their arms were fully extended [9]. The ICC of the test was 0.870.

### 2.5. Training Load 

The training camps consisted of morning and afternoon training sessions, generally lasting 5 to 6 days. The number and duration of sessions were the same for all players. Each session lasted between 90 and 120 min and included a physical training section and a technical/tactical section separated by a recovery period of ~15 min (Table 1). Quantification of the total load of each training session was determined using the session rating of perceived exertion (s-RPE), a procedure that is frequently used due to its efficiency, simplicity and reliability for monitoring and tracking athletes’ responses to training loads. Players were asked to rate their perceived exertion (RPE) on a 10-point scale, as previously proposed by Foster et al. [32], approximately 15 to 20 min after each session. Athletes were familiarized with the scale before measurements were taken. Training load (arbitrary units) = intensity (RPE) x session duration (min). Strain was then calculated as the training load multiplied by monotony (i.e., weekly).

### 2.6. Blood Samples

Analysis of blood levels of muscle damage markers (CK and LDH) and serum levels of BDNF was performed. Samples were taken in the morning between 8 and 10 a.m. after a 48 h rest and recovery period, and participants followed an overnight fast. Samples were taken by venipuncture from a superficial forearm vein and placed in 10 mL serum separator tubes (without anticoagulants). For muscle damage markers, samples were allowed to clot at room temperature (24 ± 1 °C) for 30 min, then the serum was separated by centrifugation (3000× *g* at 4 °C for 15 min) and analyzed by an automated biochemical analyzer (the results are expressed in IU/L). For BDNF, the resulting serum sample was aliquoted into 0.5 mL Eppendorf tubes and frozen at −80 °C until analysis. Concentrations (pg/mL) were determined according to the manufacturer’s instructions by an enzyme-linked immunosorbent assay (ELISSA), using R&D system kits (Human BDNF ELISA Kit, ab212166, Abcam Limited, Cambridge, UK).

### 2.7. Statistical Analyses

Statistical analyses were performed using IBM SPSS Statistics 22.0 software. Data were expressed as means and standard deviations (mean ± SD). The normal distribution of the data was verified by the Shapiro–Wilk test. The *t*-test for independent samples was used to compare the training load for the two periods (T0-T1 and T1-T2). A one-way analysis of variance (ANOVA) for repeated measures was computed to determine differences between parameters measured during the season. In the event of a significant effect, a post hoc analysis by paired *t*-test (Fisher’s protected least significant difference) was used. Comparisons between forwards and backs were made by 1-factor ANOVA. Values of *p* < 0.05 at a 95% confidence interval were considered statistically significant. Effect sizes (ESs) were calculated to compare differences in mean values for all analyzed parameters, with the following quantitative thresholds: trivial < 0.20, small 0.21–0.60, moderate 0.61–1.20, large 1.21–1.99 and very large > 2.0 [9].

## 3. Results

### 3.1. Training Load

**Figure 2:**
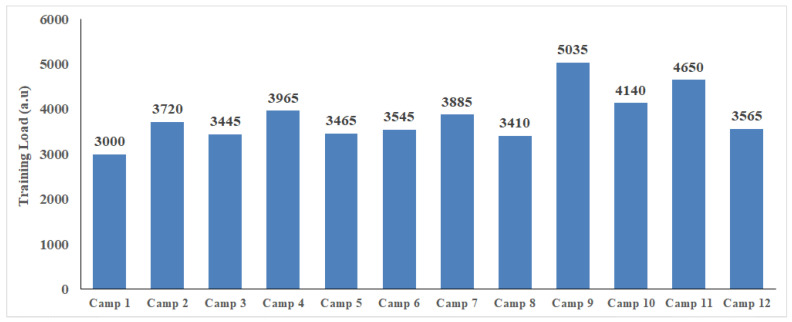
Training load per camp; a.u.: arbitrary units.

**Figure 3:**
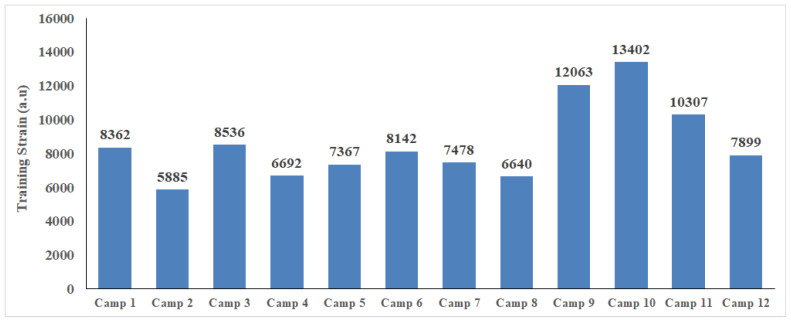
Training strain per camp; a.u.: arbitrary units.

The training loads over the season are shown in Table 2 and Figure 2. There were no differences in training load between the two evaluation periods (*p* > 0.05, Table 2). The training strain per camp is presented in Figure 3.

### 3.2. Anthropometric Characteristics

Changes in players’ anthropometric characteristics according to position are presented in Table 3. There was a decrease in % BFM for forwards between T0-T1, T0-T2 and T1-T2 (*p* < 0.05, ES_T0-T1_ = 0.36, ES_T0-T2_ = 0.64, and ES_T1-T2_ = 0.40). The forwards had higher body mass, BMI and % BFM compared to the backs at the three assessments (*p* < 0.05). There were no significant time × position interactions (*p* > 0.05).

### 3.3. Physical Fitness Testing

Analysis of the physical fitness tests indicates improved performance for forwards and backs in most tests across the evaluation sessions (*p* < 0.05, Table 4). There were no significant interactions for time × position (*p* > 0.05). When comparing the two positions, the backs had better cardiorespiratory fitness than the forwards (*p* < 0.05) and performed better in linear speed (*p* < 0.05), change-of-direction speed (*p* < 0.05) and the two jump tests (*p* < 0.05). However, the forwards performed better in the bench press test (*p* < 0.05, Table 5).

### 3.4. Muscle Damage

Monitoring variations in the muscle damage marker CK indicated decreasing levels between the beginning and end of the season for forwards (*p* < 0.05, ES_T0-T2_ = 0.82, Table 6). Levels of LDH decreased between T0-T1 and T0-T2 (*p* < 0.05, ES_T0-T1_ = 1.41, ES_T0-T2_ = 1.04) for forwards and increased between T0-T2 and T1-T2 (*p* < 0.05, ES_T0-T2_ = 2.54, ES_T1-T2_ = 1.85) for backs. There was a significant interaction for time × position (*p* = 0.001). Levels of LDH were higher at T0 for the forwards compared to the backs (*p* < 0.05). There were no significant differences at T1, but levels of CK and LDH were highest at T2 for the backs (*p* < 0.05).

### 3.5. BDNF

Variations in plasma levels of BDNF for players are shown in Table 7. Levels of BDNF decreased between the second and third sampling for forwards and backs, but the decrease was significant for the total group (*p* < 0.05, ES_T1-T2_ = 0.72). There were no differences between the two positions or time × position interaction (*p* > 0.05).

## 4. Discussion

Our study involved the longitudinal monitoring (24 weeks) of physical fitness, muscle damage and BDNF levels in rugby players during a preparatory period for an international event. The main findings of our study are that (1) there were changes in anthropometric characteristics and improvements in players’ physical fitness across the season, (2) variations in levels of two markers of muscle damage (CK and LDH) occurred between experimental periods, and (3) there were changes in serum BDNF levels for the total group.

In contemporary professional rugby, teams are confronted with many matches throughout their competitive season, including league, cup and international matches. In April 2023, the U20 African Cup Barthés Trophy was started in Kenya, with eight teams aiming to peak over this tournament. The teams will likely have used the latest performance science research to optimally prepare their athletes for this demanding competition, with the tournament structure and game intensity placing significant anthropometrical and physiological demands on the players [33]. In professional team sports, the term athlete readiness refers to the athlete’s ability to carry out training and competition activities. Optimal readiness is a state during which the athlete experiences no impairment of physical performance, excessive fatigue or psychological distress [4].

### 4.1. Anthropometric Characteristics

In our study of elite U20 rugby players, body mass and BMI did not change over 24 weeks. However, a statistical decrease in the percentage of body fat mass was observed for the forwards and the total group. Skinfolds indicate fatness located in subcutaneous storage areas and can be used to monitor changes in peripheral fat stores over time [34]. In general, elite players are heavier, have greater lean body mass and have lower skinfold and body fat percentage scores. It would appear that increased muscle mass is an important determinant of muscle strength [35]. 

Rugby differs from other sports in that the anthropometric characteristics of its players vary according to their playing position [36]. Our study shows greater body mass, BMI and %BFM in forwards than in backs during the three assessment periods. In rugby, desirable changes in body composition (increases in lean mass and/or decreases in %BFM) occur primarily when the training volume is high [37]. The decrease in %BFM between the beginning and the end of the season was ~12.35% and 14.49%, respectively, for the total group and forwards. These changes reflect small but positive adaptations in body composition and physical condition achieved during this period. These anthropometric characteristics are key factors in winning or keeping the ball and making tackles [36]. In addition, they play a protective role against the many injuries that can be caused by shocks and intense contact during the game [38]. Forwards are involved in 68% of the total collisions. Therefore, it is generally accepted that the backline players are faster, whereas the forwards have more contact and, as a result, are generally bigger and stronger players to ensure that they can handle these collisions [8]. 

### 4.2. Physical Fitness

Rugby practice requires players to possess a variety of physical fitness qualities to meet the demands of competition, such as muscular strength and power, linear and change-of-direction speed, repeated sprint ability, aerobic power and high-intensity running ability [39]. Players must be fast, powerful and agile whilst maintaining a highly developed maximal cardiorespiratory fitness [40]. The results of our physical fitness tests show an improvement in the performance of the total group and both positions.

The physiological demands of rugby practice are complex and position-specific [37]. When comparing the two positions, the backs had better cardiorespiratory fitness [7], linear sprint speed [7], change-of-direction speed [31] and lower-limb muscle power [28] performance than the forwards. The backs cover greater distances at higher speeds and perform more accelerations and decelerations than the forwards [23,24,25]. The full-back position covers the greatest total (~6800 m) and high-speed running distances (~583 m) during match play [41]. Furthermore, our results indicate that backs attained better SLJ and CMJ performances than forwards. However, the forwards were on average ~23 kg heavier than the backs (*p* < 0.05), which represented a large effect [42]. Considering these results, high-intensity and sprint training must be specific for positions [43]. Not surprisingly, the backs covered a greater distance than the forwards in the cardiorespiratory fitness tests. The result highlights the negative effect of high body mass on aerobic running test performance. The YYIRT is cited to be of sufficient sensitivity to discover training-induced changes in repeated high-intensity exercise similar to that occurring in Rugby Union [44]. 

In terms of maximal muscle strength, the results of elite U20 rugby players increased by 13.76, 10.58 and 25.02%, respectively, for squat, prone row and bench press over the preparation period (seven internships). The ability of a rugby player to express high levels of muscle strength is crucial for success in competition. However, forwards showed higher 1RM bench press values than backs (*p* < 0.05) [31]. Higher values of absolute strength and greater body mass for forwards would be favorable for this position to master contact situations [23]. Forwards are more involved in making tackles compared to backs (64% vs. 36%) [45]. There is a strong correlation between tackling ability and upper-body muscle strength, particularly in the bench press (r = 0.58, *p* < 0.01) [46]. The differences in physical fitness between the two groups in our study underline the key role of the specific physical effort of each position [47]. Individualized or position-specific training sections, with intensity, duration and type of exercise specific to the player’s profile, would appear to be relevant to maximize player performance and enable them to better meet the physical demands of their position. 

### 4.3. Muscle Damage

Previous studies have shown that the physical demands of rugby can lead to variations in muscle damage markers in the blood, whether after a 15-a-side rugby match [48], rugby league [49] match or rugby 7s’ tournament [50] or across a sporting season [17]. These variations are generally followed by a state of fatigue that can last several days and affect physical performance [14,15]. 

Our results indicate that concentrations of CK and LDH in forwards decreased between the beginning and the end of the season. For the backs, CK concentrations did not change, but LDH concentrations increased at the end of the season compared with baseline. At the start of the season, the forwards had significantly higher LDH concentrations than the backs (*p* < 0.05). At the end of the season, the highest CK and LDH concentrations were observed in the backs (*p* < 0.05).

Of note, forwards compared with backs experience significantly more tackles and collisions during match play [38,45]. These high-intensity activities during the game are associated with changes in markers of muscle damage [7]. Accordingly, we speculate that the observed differences in CK and LDH concentrations between the two positions at the end of the season could be the result of improved recovery processes in the forwards due to training-related physiological adaptations [17]. Effective monitoring strategies in rugby involve the assessment of the number and intensity of contacts during training and/or competition. This objective data can help practitioners optimize the programming of rugby training.

### 4.4. BDNF

The beneficial effects of physical activity on cognitive function and BDNF levels have received much attention in recent years and suggest a correlation between improved cognitive function and increased BDNF levels in response to physical activity, but the type and dose of physical effort to achieve optimal levels is unclear [21]. Baseline levels of BDNF in athletes were found to be higher than in sedentary people, with the highest levels observed in combat sport athletes [51]. It has been shown that strength training does not increase BDNF levels, and aerobic training may be more effective [52]. In addition, intensive training can increase BDNF levels, but this is influenced by the duration of exercise [53]. 

Our study consisted of monitoring basal BDNF levels during a sporting season in elite rugby players. Although there was no significant difference in training load between the two sampling periods, the variations in basal serum BDNF levels during these periods were different. Our results show that there were non-significant increases in BDNF levels between the first and second sampling in both groups. Levels decreased at the end of the season compared to the beginning and middle of the season for both forwards and backs, but the decrease was only significant for the total group between the second and third sampling. The results presented in the literature, as well as our results, on the dose–response relationship between physical activity and BDNF levels remains unclear. In this study, there were no significant differences in training load between the two sampling periods, but the player adaptations in each period were different. It seems interesting to compare BDNF levels from the sampling periods of different physical loads and monitor the results of questionnaires assessing cognitive abilities.

## 5. Conclusions

Physical fitness performance dominates the world of professional rugby, and the main aim of quantifying load and monitoring player reactions is to optimize training and physical preparation. Quantifying applied load is the first step in monitoring players, but quantification is only relevant when the resulting adaptations are also taken into account. The combination of objective and subjective measurements enables continuous and effective monitoring of the athlete’s response to the applied load. The monitoring of elite U20 rugby players in the present study was aimed at optimizing their preparation for the African Cup. 

Anthropometric and physical fitness characteristics are known to play a vital role in elite rugby. They vary according to position and level of play. Our study shows changes in anthropometric characteristics and improvements in physical fitness for forwards and backs throughout the season, with specificities for each group. The improvement and development of these characteristics appear to be priority objectives for the staff and managers of professional teams. It is, therefore, essential to test players regularly to assess their level and/or progress and the effectiveness of the work carried out.

Rugby is characterized by frequent contact and collision between players. This load underlies variations in muscle damage. In general, the forwards are the most stressed in the acute impact phases compared to the backs. Monitoring of muscle damage over the season showed different variations between the two groups in response to the load applied. Quantifying contact during training would appear to be an interesting way of determining the extent of the impact of this physical load on the variation in muscle damage. Understanding these effects is of great importance in informing decisions on training programming, recovery and squad management during the season. 

With the evolution of rugby and the demands of the game, cognitive abilities have become increasingly important to the success and achievement of rugby players. Cognitive function in the present study was monitored through changes in players’ BDNF levels in response to applied load over the course of the season. Although there were no differences in training load between the two sampling periods, the changes in BDNF levels over these periods were dissimilar. Despite the progress that has been made in understanding the dose–response relationship between physical activity an training with BDNF levels and cognitive function, this relationship requires further clarification. 

## 6. Study Limitations

Our study has some limitations that need to be considered: First, the sample size is rather small, and the number of measurement points is limited, which is why more research is needed to verify our findings. Second, the training load was quantified using the s-RPE. However, rugby requires different actions during match play that make the precise quantification of load difficult. Quantification of the external load using GPS data (distance covered, number of sprints, accelerations and decelerations, etc.) and the number and intensity of contacts would have been appropriate to determine more precisely the load applied to players. The combination of the two methods appears to be the most effective for monitoring load. Third, effective monitoring requires more subjective parameters, such as player fatigue and/or well-being. The use of questionnaires measuring psychometric components (sleep quality, fatigue level, muscle soreness, stress, mood, etc.) could be appropriate to monitor athletes’ response to the load applied. Finally, although BDNF is an indirect indicator of cognitive skills, combining the data obtained with the results of cognitive assessment questionnaires seems to be a more appropriate way of monitoring cognitive function.

## 7. Future Investigations

Future studies could confirm findings from our study using larger cohorts per player position. In addition, researchers could assess the effects of contact on muscle damage and neuromuscular function and use questionnaires to assess players’ well-being, fatigue and cognitive abilities. 

## Figures and Tables

**Table 1:** Example of a training camp program.

Day	Morning Session (9:30 to 11:30 AM)	Afternoon Session (3:00 to 5:00 PM)
Monday	Beginning of internship	Physical training Recovery period Technical/Tactical training
Tuesday	Physical training Recovery period Technical/Tactical training	Physical training Recovery period Technical/Tactical training
Wednesday	Rest	Rest
Thursday	Physical training Recovery period Technical/Tactical training	Physical training Recovery period Technical/Tactical training
Friday	Physical training Recovery period Technical/Tactical training	End of internship

**Table 2:** Training load per period.

	From T0 to T1	From T1 to T2	Total Preparation
Average Training Load (a.u.)	3575 ± 323.97	4160 ± 693.30	3818.75 ± 568.14
Cumulative Training Load (a.u.)	25,025	20,800	45,825

Mean ± SD reported for the average training load per period; a.u.: arbitrary units.

**Table 3:** Changes in anthropometric characteristics.

		T0	T1	T2	*p*-Value	ES _T0-T1 – T0-T2 – T1-T2_	Time × Position
Body Mass (kg)	Forwards	96.54 ± 11.56 *	97.39 ± 10.43 *	96.71 ± 9.35 *	0.652	0.07 – 0.01 – 0.07	0.579
	Backs	73.19 ± 5.65	73.14 ± 4.55	74.04 ± 4.54	0.688	0.01 – 0.15 – 0.20	
	TG	86.92 ± 15.08	87.41 ± 14.84	87.38 ± 13.75	0.773	0.03 – 0.03 – 0.01	
BMI (kg/m^2^)	Forwards	29.57 ± 3.07 *	29.82 ± 2.61 *	29.64 ± 2.48 *	0.655	0.08 – 0.02 – 0.07	0.544
	Backs	23.57 ± 1.47	23.60 ± 0.98	23.91 ± 1.32	0.563	0.03 – 0.24 – 0.32	
	TG	27.10 ± 3.92	27.26 ± 3.76	27.28 ± 3.54	0.635	0.04 – 0.05 – 0.01	
Body Fat Mass (%)	Forwards	21.12 ± 4.76 *	19.41 ± 3.77 *	18.06 ± 2.84 *	0.009 ‡£⸹	0.36 – 0.64 – 0.40	0.129
	Backs	12.68 ± 1.65	11.87 ± 1.59	11.78 ± 0.97	0.242	0.49 – 0.55 – 0.06	
	TG	17.65 ± 5.66	16.31 ± 4.69	15.47 ± 3.88	0.006 ‡£⸹	0.24 – 0.38 – 0.18	

Mean ± SD reported for forwards, backs and total group, BMI: Body mass index, TG: Total group, ES: Effect size, ‡: Significant differences between T0-T1 (*p* < 0.05), £: Significant differences between T0-T2 (*p* < 0.05), ⸹: Significant differences between T1-T2 (*p* < 0.05), *: Significant differences between forwards and backs (*p* < 0.05).

**Table 4:** Longitudinal development of cardiorespiratory fitness, linear, and change-of-direction speed, and lower limb power.

			T0	T1	T2	*p*-Value	ES _T0-T1 – T0-T2 – T1-T2_	Time × Position
		Cardiorespiratory Fitness						
YYIRT (km/h)	Forwards		14.50 ± 0.41	15.22 ± 0.43	15.70 ± 0.48	0.001 ‡£⸹	1.78 – 2.94 – 1.10	0.112
	Backs		15.43 ± 0.28 *	15.86 ± 0.24 *	16.46 ± 0.30 *	0.001 ‡£⸹	1.54 – 3.72 – 2.49	
	TG		14.88 ± 0.59	15.48 ± 0.48	16.01 ± 0.56	0.001 ‡£⸹	1.03 – 1.93 – 1.10	
		Linear Sprint Speed						
10-m (s)	Forwards		1.76 ± 0.55	1.73 ± 0.05	1.68 ± 0.05	0.001 £⸹	0.48 – 1.45 – 1.07	0.160
	Backs		1.69 ± 0.4 1*	1.61 ± 0.06 *	1.56 ± 0.07 *	0.001 ‡£⸹	1.79 – 2.96 – 0.77	
	TG		1.73 ± 0.60	1.68 ± 0.08	1.63 ± 0.08	0.001 ‡£⸹	0.76 – 1.61 – 0.63	
20-m (s)	Forwards		3.15 ± 0.12	3.15 ± 0.14	3.07 ± 0.12	0.021 £⸹	0.07 – 0.64 – 0.61	0.622
	Backs		2.97 ± 0.20 *	2.95 ± 0.11 *	2.84 ± 0.10 *	0.067	0.09 – 0.64 – 1.01	
	TG		3.07 ± 0.18	3.07 ± 0.16	2.97 ± 0.16	0.001 £⸹	0.01 – 0.56 – 0.60	
30-m (s)	Forwards		4.47 ± 0.21	4.50 ± 0.23	4.34 ± 0.14	0.015 £⸹	0.15 – 0.62 – 0.70	0.654
	Backs		4.28 ± 0.23	4.24 ± 0.13 *	4.10 ± 0.13 *	0.002 £⸹	0.14 – 0.77 – 1.08	
	TG		4.39 ± 0.23	4.40 ± 0.23	4.24 ± 0.18	0.001 £⸹	0.02 – 0.64 – 0.67	
		Change-of-Direction Speed						
*t*-test (s)	Forwards		11.02 ± 0.47	10.66 ± 0.39	10.25 ± 0.64	0.001 ‡£⸹	0.76 – 1.65 – 1.05	0.851
	Backs		9.90 ± 0.50 *	9.52 ± 0.52 *	9.21 ± 0.56 *	0.003 ‡£⸹	0.74 – 1.36 – 0.60	
	TG		10.55 ± 0.73	10.19 ± 0.72	9.82 ± 0.79	0.001 ‡£⸹	0.49 – 1.00 – 0.51	
		Lower Limb Muscle Power						
SLJ (m)	Forwards		2.07 ± 0.18	2.16 ± 0.21	2.28 ± 0.21	0.001 ‡£⸹	0.49 – 1.18 – 0.59	0.602
	Backs		2.33 ± 0.16 *	2.42 ± 0.17 *	2.51 ± 0.12 *	0.001 ‡£⸹	0.56 – 1.16 – 0.54	
	TG		2.18 ± 0.21	2.27 ± 0.23	2.38 ± 0.21	0.001 ‡£⸹	0.42 – 0.94 – 0.48	
CMJ (cm)	Forwards		29.58 ± 3.38	30.71 ± 3.54	32.10 ± 4.02	0.001 ‡£⸹	0.33 – 0.74 – 0.39	0.055
	Backs		32.79 ± 3.52	35.11 ± 3.87 *	36.71 ± 4.48 *	0.001 ‡£⸹	0.66 – 1.11 – 0.41	
	TG		30.90 ± 3.71	32.52 ± 4.20	34.00 ± 4.70	0.001 ‡£⸹	0.44 – 0.84 – 0.35	

Mean ± SD reported for forwards, backs and total group, YYIRT: Yo-Yo Intermittent Recovery Test level 1, CMJ: Countermovement jump, SLJ: Standing long jump, TG: Total group, ES: Effect size, ‡: Significant differences between T0-T1 (*p* < 0.05), £: Significant differences between T0-T2 (*p* < 0.05), ⸹: Significant differences between T1-T2 (*p* < 0.05), *: Significant differences between forwards and backs (*p* < 0.05).

**Table 5:** Longitudinal development of muscle strength.

Muscle Strength		T0	T1	*p*-Value	ES _T0-T1_
Squat (kg)	Forwards	140.10 ± 20.56	163.30 ± 20.22	0.001 ‡	1.13
	Backs	143.57 ± 11.50	157.71 ± 14.15	0.002 ‡	1.23
	TG	141.53 ± 17.04	161.00 ± 17.69	0.001 ‡	1.14
Prone Row (kg)	Forwards	148.30 ± 11.22	167.50 ± 14.24	0.001 ‡	1.71
	Backs	148.86 ± 10.95	159.57 ± 17.84	0.096	0.98
	TG	148.53 ± 10.76	164.24 ± 15.80	0.001 ‡	1.46
Bench Press (kg)	Forwards	105.90 ± 14.39 *	129.20 ± 13.94 *	0.001 ‡	1.62
	Backs	84.00 ± 17.51	109.57 ± 10.77	0.001 ‡	1.46
	TG	96.88 ± 18.84	121.12 ± 15.87	0.001 ‡	1.29

Mean ± SD reported for forwards, backs and total group, TG: Total group, ES: Effect size, ‡: Significant differences between T0-T1 (*p* < 0.05), *: Significant differences between forwards and backs (*p* < 0.05).

**Table 6:** Serum levels of CK and LDH.

Muscle Damage		T0	T1	T2	*p*-Value	ES _T0-T1 – T0-T2 – T1-T2_	Time × Position
CK (IU/L)	Forwards	771.70 ± 389.37	721.90 ± 532.42	452.80 ± 327.88	0.050 £	0.13 – 0.82 – 0.51	0.215
	Backs	927.57 ± 690.50	928.29 ± 786.47	1162.43 ± 748.11 *	0.754	0.01 – 0.34 – 0.30	
	TG	835.88 ± 519.93	806.88 ± 634.32	745.00 ± 632.41	0.902	0.06 – 0.17 – 0.10	
LDH (IU/L)	Forwards	257.10 ± 45.66 *	192.80 ± 46.62	209.70 ± 33.69	0.001 ‡£	1.41 – 1.04 – 0.36	0.001
	Backs	193.86 ± 27.05	184.29 ± 42.41	262.57 ± 58.36 *	0.003 £⸹	0.35 – 2.54 – 1.85	
	TG	231.06 ± 49.76	189.29 ± 43.77	231.47 ± 51.33	0.001 ‡⸹	0.84 – 0.01 – 0.96	

Mean ± SD reported for forwards, backs and total group, CK: Creatine kinase, LDH: Lactate dehydrogenase, TG: Total group, ES: Effect size, ‡: Significant differences between T0-T1 (*p* < 0.05), £: Significant differences between T0-T2 (*p* < 0.05), ⸹: Significant differences between T1-T2 (*p* < 0.05), *: Significant differences between forwards and backs (*p* < 0.05).

**Table 7:** Serum levels of BDNF.

BDNF		T0	T1	T2	*p*-Value	ES _T0-T1 – T0-T2 – T1-T2_	Time × Position
BDNF (pg/mL)	Forwards	3471.74 ± 1005.77	4129.72 ± 968.18	3162.01 ± 992.87	0.071	0.65 – 0.31 – 1.00	0.775
	Backs	3459.91 ± 857.66	3776.66 ± 1384.57	3188.79 ± 1124.87	0.367	0.37 – 0.32 – 0.42	
	TG	3466.87 ± 919.17	3984.34 ± 1030.60	3173.03 ± 1014.49	0.038 ⸹	0.56 – 0.32 – 0.72	

Mean ± SD reported for forwards, backs and total group, BDNF: Brain-derived neurotrophic factor, TG: Total group, ES: Effect size, ⸹: Significant differences between T1-T2 (*p* < 0.05).

## Data Availability

The raw data supporting the conclusions of this article will be made available by the authors upon request.
